# Elimination of *Arsenophonus* increases susceptibility to sulfoxaflor in *Aphis gossypii*

**DOI:** 10.3389/fmicb.2025.1708122

**Published:** 2026-01-16

**Authors:** Abulaiti Alimu, Xiao Zhong, Yu Gao, Yanhui Lu

**Affiliations:** 1 State Key Laboratory for Biology of Plant Diseases and Insect Pests, Institute of Plant Protection, Chinese Academy of Agricultural Sciences, Beijing, China; 2 Western Agricultural Research Center, Chinese Academy of Agricultural Sciences, Changji, China

**Keywords:** *Aphis gossypii*, *Arsenophonus*, P450 genes expression, RNAi, sulfoxaflor susceptibility

## Abstract

**Introduction:**

The cotton aphid, Aphis gossypii Glover, is a globally significant agricultural pest that harbors diverse microbial symbionts. Beyond their well-known roles in nutrition, these microbial partners are increasingly recognized for their potential to modulate host detoxification pathways and influence insecticide susceptibility. While sulfoxaflor is a primary insecticide for controlling A. gossypii, the extent to which the predominant secondary symbiont, Arsenophonus, mediates susceptibility to this chemical remains largely unexplored.

**Methods:**

In this study, we investigated the role of Arsenophonus in modulating host sulfoxaflor susceptibility and the underlying molecular mechanisms. We established an Arsenophonus-infected A. gossypii line (A-infected) and an antibiotic-cured, Arsenophonus-deleted line (A-deleted). To ensure identical genetic backgrounds and eliminate residual antibiotic effects, the A-deleted line was maintained for 10 generations under antibiotic-free conditions, with symbiont status confirmed by PCR and 16S rRNA sequencing. We then compared sulfoxaflor susceptibility, analyzed protein levels of detoxification enzymes, performed comparative transcriptomic analysis, and validated key candidate genes using RNA interference (RNAi).

**Results and discussion:**

Bioassays revealed that the elimination of Arsenophonus significantly increased susceptibility to sulfoxaflor. This hypersensitivity was metabolically associated with reduced protein levels of mixed-function oxidases (MFOs) and glutathione S-transferases (GSTs). Comparative transcriptomic analysis identified multiple differentially expressed cytochrome P450 genes, including *CYP380C44*, *CYP380C45*, *CYP6J1*, *CYP6CY14*, *CYP6CY21*, *CYP4CJ1*, and *CYP4C1*. Functional verification demonstrated that RNAi-mediated silencing of *CYP380C44* in the A-infected line significantly increased sulfoxaflor mortality. Collectively, our findings demonstrate that the secondary symbiont Arsenophonus modulates the host response to sulfoxaflor by regulating P450-mediated metabolic pathways. Identifying *CYP380C44* as a critical effector gene highlights the Arsenophonus-P450 axis as a potential molecular target for developing novel pest control strategies that exploit symbiotic vulnerabilities

## 1 Introduction

Nearly all insects harbor endosymbionts, fostering intricate co-evolutionary dynamics. These ubiquitous microbial partners play pivotal roles in host survival, fitness, and ecological adaptability (Kaltenpoth et al., 2025). Symbiotic bacteria regulate host nutritional and reproductive metabolism by provisioning essential amino acids, and vitamins. Beyond nutritional supplementation, defensive symbionts confer protection against various environmental challenges, including thermal stress (Russell and Moran, 2006; Zhang et al., 2019), natural enemies, and fungal pathogens (Łukasik et al., 2013; Oliver et al., 2003; Oliver et al., 2010). Crucially, recent evidence highlights their role in enhancing host tolerance to insecticides (Kikuchi et al., 2012; Lv et al., 2023; Zeng et al., 2023).

As one of the world’s largest producers and consumers of pesticides, China remains heavily dependent on chemical interventions for agricultural pest management (Wu et al., 2021). Consequently, the intensification of chemical usage has precipitated rising insecticide resistance, which has emerged as a critical constraint on sustainable agriculture and environmental safety (Souto et al., 2021). Traditionally, insecticide resistance has been ascribed to host-encoded genetic adaptations, primarily target-site insensitivity and metabolic sequestration driven by selection pressure (Bass and Nauen, 2023; Bass et al., 2014; Liu et al., 2025; Liu and Guo, 2019). However, accumulating evidence indicates that symbiotic microbiota also playing a pivotal role in modulating susceptibility (Cheng et al., 2017; Li et al., 2021; Malook et al., 2025; Zhang et al., 2025). Consequently, symbiont-mediated detoxification is increasingly recognized as a critical “third resistance mechanism,” functioning alongside host intrinsic defenses (Sato et al., 2021; Blanton and Peterson, 2020).

The interaction between symbiotic bacteria and insecticide resistance exhibits highly context-dependent, governed by the specific interplay of symbiont strain, host identity, and insecticide class (Pang et al., 2018; Sato et al., 2021; Malook et al., 2025). For instance, in the pea aphid, *Acyrthosiphon pisum*, infection with *Serratia symbiotica* actually increases sensitivity to certain insecticides, likely due to the severe fitness costs that compromise host development, reproduction, and body weight (Skaljac et al., 2018). Whereas in the brown planthopper, *Nilaparvata lugens*, the impact is highly strain-specific; replacing the resident *Arsenophonus* N-type strain with the S-type significantly reduces resistance (Pang et al., 2018). Conversely, antibiotic suppression of bacterial symbionts in *N. lugens* increases susceptibility to imidacloprid, chlorpyrifos, and clothianidin by suppressing P450 and GST activities and downregulating genes such as *NlCYP6ER1* and *NlCYP4CE1* (Tang et al., 2021). Furthermore, *Arsenophonus* and *Wolbachia* infections in *N. lugens* enhance protection against triflumezopyrim and dinotefuran, with *Wolbachia* specifically linked to the upregulation of *GSTm2* and *CYP6AY1* (Liu et al., 2025). A similar pattern is observed in the whitefly *Bemisia tabaci*, where *Arsenophonus* and *Wolbachia* abundance correlates with resistance to neonicotinoids, tracking with the upregulation of specific P450 genes (e.g., *CYP6CM1*, *CYP6DZ7*, *CYP6CX1*), and suggesting a model in which symbionts and host detoxification enzymes co-mediate resistance (Ghanim and Kontsedalov, 2009; Barman et al., 2021; Barman et al., 2022).

The cotton aphid, *Aphis gossypii* Glover (Hemiptera: Aphididae), is a major global agricultural pest causing substantial economic losses through direct feeding and virus transmission (Bass et al., 2015; Wu and Guo, 2005). Management of *A. gossypii* relies predominantly on chemical insecticides, with sulfoxaflor acting as a key control agent. However, intensive and prolonged use of sulfoxaflor and other insecticides has led to varying levels of resistance in field populations (Shang et al., 2012). Despite growing evidence for symbiont-associated resistance in other hemipterans, the contribution of symbionts to insecticide susceptibility in *A. gossypii* remains poorly understood. Previous studies have shown that resistant strains exhibit altered microbiome compositions, with notably higher abundances of *Arsenophonus* compared with susceptible strains (Zhang et al., 2016), and that sublethal sulfoxaflor exposure can increase the relative abundance of *Arsenophonus* in subsequent generations (Shang et al., 2021). However, the specific role of *Arsenophonus* in modulating sulfoxaflor susceptibility and the underlying molecular mechanisms in *A. gossypii* remain unclear.

In this study, to examine the specific contribution of the facultative symbiont *Arsenophonus* to sulfoxaflor susceptibility in *A. gossypii* and explored the associated molecular mechanisms. To this, we established a natural *Arsenophonus*-infected line (A-infected) and an antibiotic-cured, *Arsenophonus*-free line (A-deleted). Our results showed that the elimination of *Arsenophonus* significantly enhanced host sensitivity to sulfoxaflor, accompanied by reduced enzymatic activities of glutathione S-transferases (GSTs) and mixed-function oxidases (MFO). Transcriptomic analysis further revealed that the absence of *Arsenophonus* led to the downregulation of the P450 pathway and diminished detoxification capacity, with *CYP380C44* identified as a core gene driving this symbiont-mediated tolerance.

## 2 Materials and methods

### 2.1 Aphid culture and establishment of isofemale lines

A field population of *A. gossypii* naturally infected with *Arsenophonus* was collected from cotton plants at the Korla Experimental Base, Chinese Academy of Agricultural Sciences (CAAS), Xinjiang, in 2022 (Alimu et al., 2025). The aphids were transferred to insect-free cotton plants and reared in nylon-mesh cages (40 cm × 40 cm × 60 cm) within a climate-controlled chamber (26 ± 1 °C, 60 ± 5% RH, 16:8 h L:D). To establish isofemale lines, apterous adult aphids were individually reared in 12-well cell culture plates. Each well contained a fresh cotton leaf disc placed adaxial side up on a 1% agar substrate. The plates were sealed with 100-mesh nylon netting and incubated in a growth chamber under the standard conditions described above. Leaf discs and agar were replaced every 2–3 days. After 15 days, genomic DNA was extracted from the offspring of each foundress. PCR screening was performed using *Arsenophonus*-specific primers (Table 1), with *Buchnera* serving as a positive control. A line confirmed positive for *Arsenophonus* was retained, propagated, and designated as the *Arsenophonus*-infected line (A-infected).

**Table 1:** Specific primers used for *Buchnera* and *Arsenophonus* in this study.

Symbionts	Target gene	Primer sequence (5′–3′)	Product (bp)
*Buchnera*	dnaK	GGTAGAATTGCTGGTTTAGAAGT	770
		TTGCTGATAAGTGAAGTCATTACA	
*Arsenophonus*	16S rRNA	AGAGTTTGATCMTGGCTCAG	960
		TTAGCTCCGGAGGCCACAGT	

### 2.2 Antibiotic treatment and establishment of the *Arsenophonus*-deleted line

To establish an *Arsenophonus*-free isofemale line (A-deleted) which has the same genetic background as the A-infected line, a dietary antibiotic treatment was administered following the protocol of Tian et al. (2023) with slight modifications. An artificial diet was prepared by mixing ampicillin (400 μg/mL), gentamicin (200 μg/mL), and cefotaxime (200 μg/mL) with 20% (w/v) sucrose solution in a 1:1:1:1 volumetric ratio.

The feeding assay utilized glass tubes (3 cm length, 2 cm inner diameter) open at both ends. A diet sachet was constructed at one end using two layers of UV-sterilized Parafilm M encapsulating150 μL of the antibiotic diet. Twenty third-instar–instar nymphs from the A-infected line were introduced into each tube, and the open end was sealed with 100-mesh nylon netting. To induce feeding via positive phototaxis, tubes were wrapped in black cloth, leaving only the diet sachet exposed to light. Three biological replicates were conducted, with an antibiotic-free 20% sucrose solution serving as the control. After 72 h, surviving nymphs were individually transferred to fresh cotton leaf discs. The resulting offspring were screened via PCR, and individuals testing negative for *Arsenophonus* were selected. To ensure the elimination of residual antibiotic effects and prevent symbiont recrudescence, the A-deleted line was reared under antibiotic-free conditions for at least 10 generations prior to subsequent experiments, with PCR verification conducted each generation.

### 2.3 Validation via 16S rRNA amplicon sequencing

To further validate the efficacy of antibiotic-mediated *Arsenophonus* elimination, 16S rRNA amplicon sequencing targeting the V3–V4 region was performed on both A-infected and A-deleted lines. For each line, five biological replicates were collected, with each replicate consisting of 20 apterous adults preserved in 1.0 mL absolute ethanol. Total genomic DNA was extracted, and the hypervariable V3–V4 region was amplified using primers 338F (5′ACTCCTACGGGAGGCAGCA-3′) and 806R (5′-GGACTACHVGGGTWTCTAAT-3′) (Yu et al., 2005). The resulting amplicons were purified and used for library construction, followed by paired-end sequencing (2 × 250 bp) on an Illumina NovaSeq 6000 platform (Beijing Biomarker Biotechnology Co., Ltd., Beijing, China).

### 2.4 Sulfoxaflor susceptibility bioassay

Sulfoxaflor toxicity to *A. gossypii* was evaluated via a leaf dip bioassay method (Mostafiz et al., 2019). Sulfoxaflor (99.99% purity) was provided by Dr. Li Cui (Institute of Plant Protection, CAAS). A stock solution (10,000 mg/L) was prepared in dimethyl sulfoxide (DMSO) and serially diluted with 0.05% (v/v) Triton X-100 water solution to generate 6–7 geometrically spaced concentrations. The control group was treated with 0.05% Triton X-100 containing an equivalent concentration of DMSO. Fresh cotton leaf discs (23 mm diameter) were dipped in the test solutions for 15 s, air-dried in the shade, and placed abaxial side up onto 1.5% agar in 12-well plates.

For the bioassay, 10 1-day-old apterous adults from either the A-infected or A-deleted line were introduced onto each leaf disc. The wells were sealed with breathable paper to prevent escape. Each concentration included five biological replicates, with 60 aphids per replicate (totaling 300 aphids per concentration). All bioassays were conducted in a climatic chamber at 26 ± 1 °C, 60 ± 5% RH, and a 16:8 h (L:D) photoperiod. Mortality was assessed after 48 h; aphids were considered dead if they failed to move their legs or antennae when gently prodded with a fine brush.

Mortality data were corrected for control mortality using Abbott’s formula (Abbott, 1925). The median lethal concentration (LC_50_), as well as LC_25_, and LC_75_ values, along with their 95% confidence intervals (CIs), were calculated using Probit analysis in PoloPlus 2.0. The goodness-of-fit for the dose–response regression lines was evaluated using the chi-square (𝜒^2^) test.

### 2.5 Quantification of detoxification and target enzyme levels

To investigate metabolic resistance mechanisms, 1-day-old apterous adults from both A-infected and A-deleted lines were exposed to sulfoxaflor at the LC_50_ dose determined for the A-infected line (50.06 mg L^−1^). Surviving aphids were sampled at 24 and 48 h post-exposure. For each time point, 20 aphids were pooled into a 1.5 mL RNase-free microcentrifuge tube, serving as one biological replicate. Three biological replicates were established for each line at each time point. Samples were immediately flash-frozen in liquid nitrogen and stored at −80 °C until analysis.

The protein levels of three key detoxification enzymes, glutathione S-transferases (GSTs), carboxylesterases (CarE), and mixed-function oxidases (MFOs), as well as the target enzyme acetylcholinesterase (AChE), were quantified using insect-specific ELISA kits (Shanghai Coibo Biotechnology Co., Ltd., Shanghai, China) according to the manufacturer’s instructions.

### 2.6 Transcriptome sequencing and differential expression analysis

#### 2.6.1 Sample collection

Samples were collected from both A-infected and A-deleted lines. Three biological replicates were established for each line, with each replicate consisting of a pool of 30 1-day-old apterous adult aphids. All samples were placed in labeled 1.5 mL RNase-free microcentrifuge tubes, immediately flash-frozen in liquid nitrogen, and stored at −80 °C.

#### 2.6.2 RNA extraction, library construction, and sequencing

RNA extraction, library preparation, and sequencing (Illumina NovaSeq 6000, PE150 mode) were conducted by Beijing Biomarker Biotechnology Co., Ltd. (Beijing, China). Raw data were processed to yield clean reads by removing adapters and low-quality sequences (criteria: N content >10% or >50% of bases with *Q* ≤ 10).

#### 2.6.3 Read alignment and gene quantification

Clean reads were aligned to the *A. gossypii* reference genome (*Aphis_gossypii*. ASM401081v1. genome.fa), using HISAT2. Transcript assembly and quantification were performed with StringTie, and gene-level read counts were obtained with feature counts. Differential expression analysis was performed using DESeq2-edgeR package. While *p*-values were adjusted for multiple testing using the Benjamini-Hochberg procedure to calculate the false discovery rate (FDR), this strict correction proved overly conservative for our specific dataset, potentially masking biologically relevant candidates (e.g., P450 family genes). Therefore, to minimize false negatives (Type II errors) in this exploratory analysis, differentially expressed genes (DEGs) were identified based on a combined threshold of a *p*-value <0.05 and a fold change (FC) ≥1.5. To ensure the reliability of this screening strategy, the expression profiles of key candidate genes identified under these criteria were subsequently validated by RT-qPCR, which demonstrated high consistency with the RNA-seq results. Finally, KEGG pathway enrichment analysis was performed on the identified DEGs, with pathways considered significantly enriched at adjusted *p*-values <0.05.

### 2.7 RT-qPCR validation

Eight cytochrome P450 (CYP450) genes were selected from the DEGs for validation, with *β-actin* served as the reference gene. Primers were designed with NCBI Primer-BLAST based on sequences obtained from the RNA-seq data, targeting amplicon sizes of 100–250 bp. cDNA synthesis was performed using the same RNA samples used for RNA sequencing. First-strand cDNA was synthesized from 2 μg of total RNA (the same samples used for sequencing) using the FastKing One-Step RT-gDNA Clean Kit (TIANGEN Biotech, Beijing, China) according to the manufacturer’s protocol, which included a genomic DNA removal step.

RT-qPCR was performed using SuperReal PreMix Plus (SYBR Green; TIANGEN) on an ABI 7500 Real-Time PCR System. Each 20 μL reaction contained 10 μL of 2 × Master Mix, 0.6 μL of each gene-specific primer (10 μM), 2 μL of cDNA template, 0.4 μL of 50 × ROX Reference Dye, and RNase-free water. The thermal cycling conditions were: initial denaturation at 95 °C for 15 min, followed by 40 cycles of 95 °C for 10 s and 60 °C for 32 s (data acquisition). A melt curve analysis was performed immediately after amplification to verify primer specificity. Three biological replicates (each with three technical replicates) were analyzed per gene. The relative expression levels were calculated using the 2^−ΔΔCT^ method (Livak and Schmittgen, 2001). All primers used in this study are listed in Table 2.

**Table 2:** RT-PCR primers used in this study.

Gene ID	Primer sequence (5′–3′)	Product (bp)
gene-LOC114121962	F: AGGTTCAATGCGTCAAGGGG	200
	R: GACTGACACCGTGCTGGTGA	
gene-LOC114131539	F: CTGTCGACTTCATCTGCCGT	173
	R: AGGACATCCTTGACGTTGCC	
gene-LOC114125146	F: AACCACGGTGACAATAAGCCT	197
	R: ACCTTGTCTTGAATTTTTGGATGG	
gene-LOC114131820	F: GACTGGACCGCCGTCATAAA	160
	R: TGGCTTGCCTATGGTTACGG	
gene-LOC114131834	F: GACTGGACCGCCGTCATAAA	160
	R: TGGCTTGCCTATGGTTACGG	
gene-LOC114124336	F: GTTAGCAACGCTCGAACACC	109
	R: GATCATCAGGAACGGCGTCT	
gene-LOC114121695	F: TCGTATTTCACCGACCACGG	180
	R: TGTCAACTGATCGCTGCACT	
β-actin	F: TGACTTGACCGACTACTTGATG	117
	R: TCCAAAGCGACATAGCACAA	

### 2.8 dsRNA synthesis and purification

Primers targeting the *CYP380C44* (LOC114125146) and the control *GFP* (pEGFP-N1, GenBank U55762.1) were designed using the FlyRNAi tool and Geneious and T7 promoter sequences were appended to the 5′ ends of both forward and reverse primers (Table 3). Template cDNA was amplified using 2 × Taq Master Mix (Vazyme, Nanjing, China) in a 50 μL reaction system containing 25.0 μL Master Mix, 2.0 μL primer mixture (10 μM each), 4.0 μL cDNA, and 19.0 μL RNase-free water. The thermal cycling conditions were as follows: initial denaturation at 95 °C for 5 min; 34 cycles of 95 °C for 30 s, 60 °C for 30 s, and 72 °C for 45 s; followed by a final extension at 72 °C for 10 min. The PCR products were purified using the MagBeads PCR Cleanup Kit (Belong Bio, Shanghai, China) following the manufacturer’s detailed protocols.

**Table 3:** Primers used for RNAi.

Gene	Primer (5′–3′)	Product
*CYP380C44*	F: taatacgactcactatagggACAACAGTGCAAATATTGAAGCG	502 bp
	R: taatacgactcactatagggTCAAGAACATTGGCGTTAAAGAC	502 bp
*GFP*	F: taatacgactcactatagggGACGTAAACGGCCACAAGTT	496 bp
	R: taatacgactcactatagggTGTTCTGCTGGTAGTGGTCG	496 bp

Double-stranded RNA (dsRNA) synthesis and purification were performed using the T7 RNA Transcription Kit Plus and MagBeads dsRNA Purification Kit (Belong Bio, Shanghai, China), following the manufacturer’s detailed protocols. Briefly, 1 μg of purified linear DNA template was combined with T7 RNA Polymerase Mix and NTP Mix, and the reaction was incubated at 37 °C for 4 h. To promote dsRNA formation, the reaction was incubated at 72 °C for 10 min and then cooled to room temperature for 20 min. Residual DNA templates were removed by digestion with RNase-free DNase I (Belong Bio, Shanghai, China) at 37 °C for 15 min. The synthesized dsRNA was purified using the MagBeads dsRNA Purification Kit (Belong Bio, Shanghai, China) to eliminate free nucleotides and proteins. The integrity of the dsRNA was verified by 1% agarose gel electrophoresis, and its concentration was quantified using a NanoDrop 2000 spectrophotometer (Thermo Fisher Scientific, Wilmington, DE, United States).

### 2.9 Dietary RNAi and bioassay

The artificial diet feeding protocols were adapted from Mittler and Dadd (1964) with minor modifications. To prevent RNase contamination, all diet components were prepared using DEPC-treated water. The basal diet consisted of 5% (w/v) sucrose. For RNAi treatments, the basal diet was supplemented with dsRNA-CYP380C44 to a final concentration of 150 ng/μL. One-day-old adult aphids were transferred to the feeding chambers and maintained under controlled conditions at 26 ± 1 °C, 60 ± 5% relative humidity (RH), and a 16:8 h (L:D) photoperiod. To evaluate RNAi efficiency, aphids were sampled at 24, 48, and 72 h post-feeding. Samples were immediately flash-frozen in liquid nitrogen and stored at −80 °C. Total RNA was subsequently extracted and reverse-transcribed, and the relative mRNA expression levels of *CYP380C44* were quantified via RT-qPCR. Three biological replicates were analyzed for each time point, with 15 adults per replicate.

Based on the RNAi efficiency result, a 48 h post-feeding interval was selected for the sulfoxaflor susceptibility bioassays. Three dietary treatments were established: (i) 5% (w/v) sucrose containing dsRNA-CYP380C44 at 150 ng/μL, (ii) 5% (w/v) sucrose containing dsGFP at 150 ng/μL (negative control), and (iii) 5% (w/v) sucrose alone (blank control). At 48 h after dietary exposure, aphids were treated with sulfoxaflor at a concentration of 50.055 mg/L, as described in Section 2.3. Mortality was assessed 48 h after insecticide application. Each treatment included three biological replicates, with 30 adults per replicate.

### 2.10 Statistical analysis

Statistical analyses were performed using GraphPad Prism 9. Prior to analysis, data assumptions were rigorously verified. Normality was assessed using the Shapiro–Wilk test, and homogeneity of variance was evaluated using the *F*-test. Comparisons between two groups were conducted based on these pre-tests: datasets meeting both normality and homogeneity of variance assumptions were analyzed using the unpaired Student’s *t*-test; datasets with normal distribution but unequal variances were analyzed using Welch’s *t*-test; and datasets that failed the normality assumption were analyzed using the non-parametric Mann–Whitney *U* test.

## 3 Results

### 3.1 Antibiotic treatment specifically eliminates *Arsenophonus* without disrupting the commensal community

**Figure 1:**
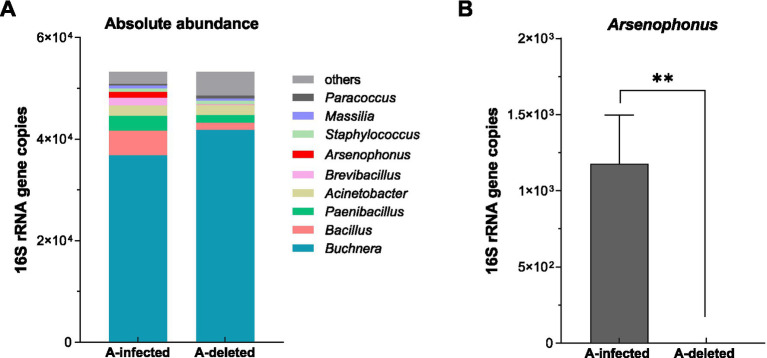
Quantification of bacterial symbionts in *A. gossypii*. **(A)** 16S rRNA gene copy numbers of the top 10 genera. **(B)** Specific titer of *Arsenophonus* in A-infected versus A-deleted lines. Bars represent mean ± SEM. Statistical comparisons were performed via an unpaired *t* test, * indicates *p* < 0.05, ** indicates *p* < 0.01 and ns indicates no significant correlation.

To validate the PCR results and assess the impact of antibiotic treatment on the broader commensal community, we performed 16S rRNA amplicon sequencing on both A-infected and A-deleted lines. We compared the abundance of the top 10 dominant bacterial genera between the two lines (Figure 1A). Consistent with previous screening, *Arsenophonus* was undetectable in the antibiotic-treated A-deleted line, showing a significant reduction compared to the A-infected line (*t* = 3.684, df = 8, *p* = 0.006; Figure 1B) confirming effective elimination. Crucially, the levels of the primary symbiont *Buchnera* remained stable and did not differ significantly between the lines (*p* > 0.05). Although other dominant genera, including *Bacillus*, *Paenibacillus*, *Brevibacillus*, and *Massilia*, exhibited a slight decreasing trend in the A-deleted line, these variations were not statistically significant (*p* > 0.05).

Collectively, these results confirm that the antibiotic regimen successfully removed *Arsenophonus* without significantly disrupting *Buchnera* or other major commensal bacteria.

### 3.2 Elimination of *Arsenophonus* increases the susceptibility of *Aphis gossypii* to sulfoxaflor

The toxicity of sulfoxaflor on A-infected and A-deleted lines was assessed using the leaf dip method, and 48 h mortality data were analyzed using Poloplus2 software (Table 4). The A-infected line exhibited LC_25_, LC_50_, and LC_75_ values of 5.00 mg/L (95% CI: 1.40–10.94), 50.06 mg/L (95% CI: 28.25–74.43) and 501.44 mg/L (95% CI: 348.71–827.82), respectively. In contrast, the A-deleted line showed increased sensitivity, with corresponding values of 2.27 mg/L (95% CI: 0.17–7.68), 20.82 mg/L (95% CI: 5.47–41.32), and 191.10 mg/L (95% CI: 118.25–326.87).

**Table 4:** Toxicity parameters of sulfoxaflor against A-infected and A-deleted *A. gossypii* lines.

Isofemale lines	*N*	Slope ± SE	LC_25_ (mg/L)	LC_50_ (mg/L)	LC_75_ (mg/L)	𝜒^2^ (df)
			95% CI	95% CI	95% CI	
A-infected	2,067	0.674 ± 0.052	5.00 (1.40–10.94)	50.06 (28.25–74.43)	501.44 (348.71–827.82)	8.04 (5)
A-deleted	2,173	0.701 ± 0.053	2.268 (0.17–7.68)	20.820 (5.47–41.32)	191.10 (118.25–326.87)	18.10 (5)

To quantify the change in susceptibility, resistance ratios (RR= LC_A-infected_/LC_A-deleted_) were calculated. The A-deleted line was consistently more sensitive to sulfoxaflor across all lethality levels compared to the A-infected line, yielding RRs of 2.21, 2.40, and 2.62 at the LC_25_, LC_50_, and LC_75_ levels, respectively. These results indicate that the elimination of *Arsenophonus* increased *A. gossypii* sensitivity to sulfoxaflor.

### 3.3 *Arsenophonus* elimination reduces protein levels of MFO and GSTs

**Figure 2:**
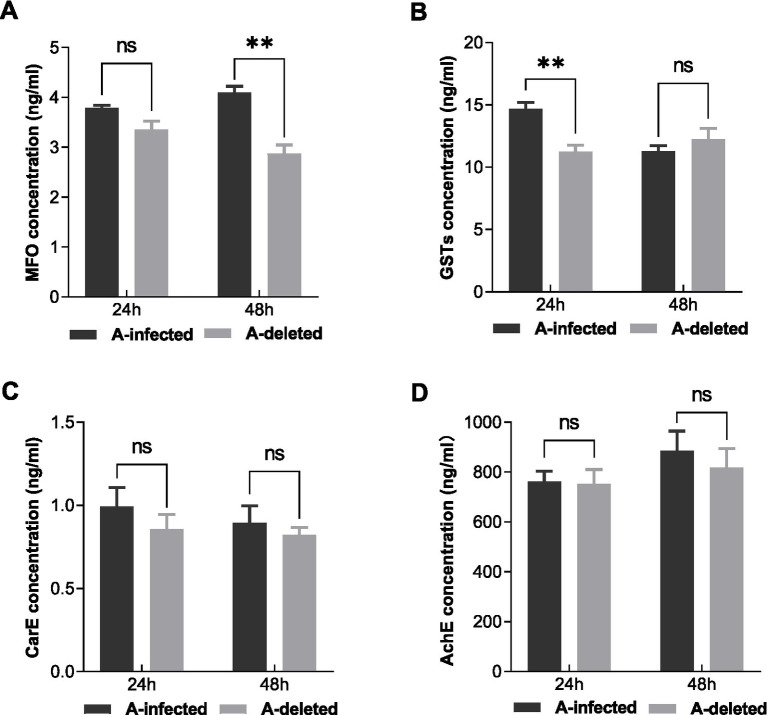
Protein levels of detoxification enzymes (MFO, CarE, and GST), and AChE in A-infected and A-deleted lines at 24 h and 48 h after LC_50_ sulfoxaflor exposure: **(A)** AChE, **(B)** MFO, **(C)** CarE, **(D)** GSTs. Bars represent mean ± SEM. Statistical comparisons were performed via an unpaired *t* test, * indicates *p* < 0.05, ** indicates *p* < 0.01 and ns indicates no significant correlation.

To investigate the mechanisms underlying altered susceptibility, we quantified the protein levels of AChE, MFO, CarE, and GSTs in both lines using ELISA. Measurements were taken at 24 and 48 h following exposure to the sulfoxaflor LC_50_ (Figure 2). At 24 h post-treatment, although AChE, MFO, and CarE levels showed no statistically significant differences between the two lines, GSTs protein levels were significantly reduced in the A-deleted line. Specifically, GSTs decreased by approximately 23.3% in the A-deleted line (11.26 ± 0.51 ng/mL) compared to the A-infected line (14.68 ± 0.51 ng/mL) (*t* = 4.755, df = 4.000, *p* = 0.009). At 48 h, a significant difference was observed in MFO levels. The A-deleted line exhibited a 29.8% reduction in MFO protein content (2.88 ± 0.17 ng/mL) compared to the A-infected line (4.10 ± 0.13 ng/mL) (*t* = 5.803, df = 3.676, *p* = 0.006). The levels of other enzymes (AChE, CarE, and GSTs) did not differ significantly between the lines at this time point.

These results indicate that the antibiotic-mediated elimination of *Arsenophonus* is associated with significantly downregulated protein levels of specific detoxifying enzymes, notably GSTs and MFO, in *A. gossypii*.

### 3.4 Transcriptomic profiling reveals downregulation of P450 metabolic pathways

**Figure 3:**
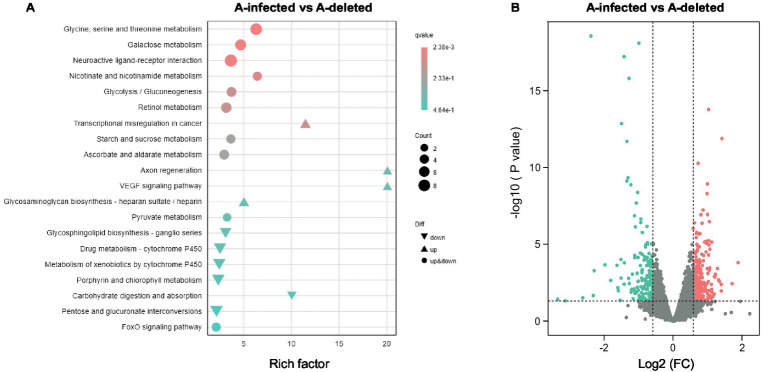
Transcriptomic analysis of *A. gossypii* in A-infected and A-deleted lines. **(A)** KEGG pathway enrichment analysis of differentially expressed genes (DEGs). **(B)** Volcano plot of DEGs.

High-throughput sequencing on the Illumina NovaSeq 6000 platform yielded a total of 37.94 Gb of clean data for the A-infected and A-deleted lines, averaging 5.76 Gb per sample. The sequencing quality was high, with Q30 scores exceeding 98.72% for all samples. Clean reads were aligned to the *A. gossypii* reference genome (ASM401081v1) with mapping efficiencies ranging from 88.93 to 92.99%, confirming the high reliability of the dataset for downstream analysis. Differential expression analysis identified a total of 404 differentially expressed genes (DEGs), comprising 147 upregulated and 257 downregulated genes (Figure 3 and Supplementary Table S1). These results indicate that the antibiotic-mediated removal of *Arsenophonus* resulted in significant alterations to the host transcriptomic profile.

To elucidate the molecular mechanisms underlying the altered sulfoxaflor susceptibility, we performed KEGG pathway enrichment analysis on the identified DEGs (Figure 3). This analysis highlighted two prominent pathways related to xenobiotic metabolism: “drug metabolism—cytochrome P450” and “metabolism of xenobiotics by cytochrome P450.” Notably, the majority of genes assigned to these pathways were downregulated in the A-deleted line. Collectively, these findings suggest that the elimination of *Arsenophonus* is associated with the downregulation of key genes involved in P450-mediated detoxification. This suppression likely contributes to impaired sulfoxaflor metabolism, thereby increasing the susceptibility of the A-deleted line.

### 3.5 Validation of P450 gene expression via RT-qPCR

Converging evidence from prior detoxification enzyme assays and KEGG pathway enrichment indicates that cytochrome P450 (CYP)-mediated detoxification is the principal molecular correlate of the greater sulfoxaflor susceptibility observed in the A-deleted strain. Compared with A-infection, RNA-seq identified seven differentially expressed CYP genes in the A-deleted group (Table 5).

**Table 5:** Statistical summary of cytochrome P450 DEGs.

Gene ID	Gene name	Up/Down	Fold change	Description
gene-LOC114125146	*CYP380C44*	Down	−1.306	Cytochrome P450 4C1-like isoform X1
gene-LOC114131820	*CYP380C45*	Down	−0.903	Cytochrome P450 4C1-like
gene-LOC114131834	*CYP4C1*	Down	−0.848	Cytochrome P450 4C1-like
gene-LOC114131539	*CYP6J1*	Down	−0.692	Cytochrome P450 6j1-like
gene-LOC114121695	*CYP6CY14*	Up	0.850	Probable cytochrome P450 6a14
gene-LOC114124336	*CYP6CY21*	Up	0.749	Probable cytochrome P450 6a13
gene-LOC114121962	*CYP4CJ1*	Up	0.647	Cytochrome P450 4C1-like

**Figure 4:**
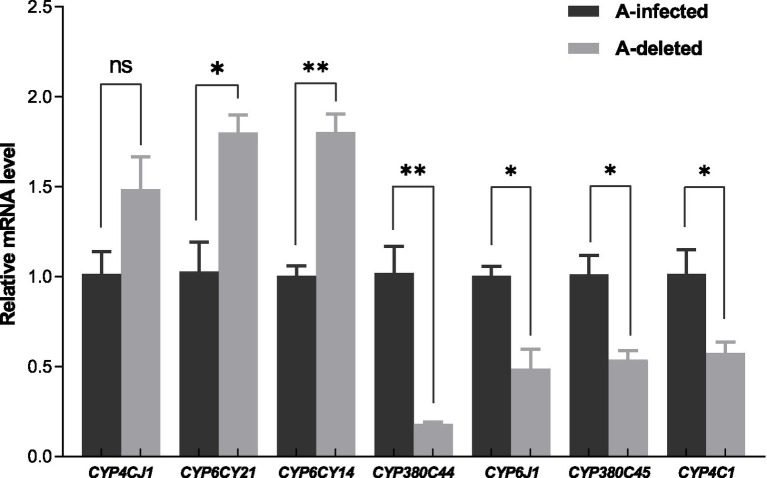
RT-PCR validation of seven selected DEGs identified via RNA-Seq. Bars represent mean ± SEM. Statistical comparisons were performed via an unpaired *t* test, * indicates *p* < 0.05, ** indicates *p* < 0.01 and ns indicates no significant correlation.

To validate the RNA-seq results, these seven DEGs were assessed via RT-qPCR (Figure 4), and contrasting results were observed. Expression of *CYP4CJ1* (*t* = 2.152, df = 3.553, *p* = 0.1064) was unaffected. By contrast, *CYP380C44* (*t* = 5.66, df = 4, *p* = 0.0049), *CYP380C45* (*t* = 4.019, df = 4, *p* = 0.0159), *CYP6J1* (*t* = 4.287, df = 4, *p* = 0.0128), *CYP6CY14* (*t* = 6.916, df = 4, *p* = 0.0023), *CYP6CY21* (*t* = 4.080, df = 4, *p* = 0.0151), and *CYP4C1* (*t* = 2.979, df = 4, *p* = 0.04408) were significantly altered, with *CYP380C44* and *CYP6CY14* showing the strongest effects (*p* < 0.01). The direction and magnitude of expression changes were concordant between RT-qPCR and RNA seq, suggesting that the absence of *Arsenophonus* modulates the expression of multiple P450 genes in *A. gossypii* and verifies the reliability of the RNA seq dataset.

### 3.6 Silencing of *CYP380C44* increases sulfoxaflor susceptibility

**Figure 5:**
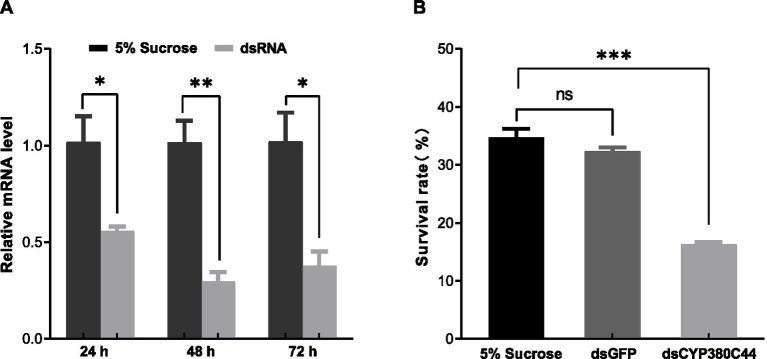
Functional verification of *CYP380C44* using RNA interference (RNAi). **(A)** Silencing efficiency of *CYP380C44* at 24, 48, and 72 h post-treatment. **(B)** Impact of *CYP380C44* silencing on the survival of *A. gossypii* exposed to sulfoxaflor (LC_50_) for 48 h. Bars represent mean ± SEM. Statistical comparisons were performed via an unpaired *t* test, * indicates *p* < 0.05, ** indicates *p* < 0.01 and ns indicates no significant correlation.

Guided by RNA-seq and RT-qPCR, which revealed that *CYP380C44* was the most strongly shifted P450 DEG and confirmed the relationship between the downregulation of *CYP380C44* and sulfoxaflor sensitivity, *CYP380C44* mRNA was reduced by RNAi in the A-infected line, with a maximal reduction of 71.01% at 48 h (45.30% at 24 h and 63.00% at 72 h, Figure 5). At the sulfoxaflor LC_50_ (50.06 mg L^−1^), mortality increased to 83.73% after dsCYP380C44 treatment compared with 67.66% (dsGFP) and 65.36% (5% sucrose) (Figure 5), indicating that *CYP380C44* contributes to sulfoxaflor detoxification in *A. gossypii*.

## 4 Discussion

### 4.1 Protective role of *Arsenophonus* against sulfoxaflor toxicity

Our results demonstrate a negative association between the facultative symbiont *Arsenophonus* and sulfoxaflor susceptibility in *A. gossypii*. The antibiotic-mediated elimination of *Arsenophonus* (A-deleted line) resulted in significantly greater susceptibility to sulfoxaflor compared to the infected line (A-infected). These observations align with previous findings in aphids, where *Arsenophonus* abundance correlates with increased fitness under insecticide pressure (Shang et al., 2021) and increased abundance of *Arsenophonus* was detected in resistant versus susceptible strains (Zhang et al., 2016). However, symbiont effects are context-dependent. For instance, in *Nilaparvata lugens* and *Bemisia tabaci*, *Arsenophonus* infection can sometimes increase insecticide susceptibility depending on the specific insecticide and co-infecting partners (Cai et al., 2024; Ghanim and Kontsedalov, 2009). Collectively, our data reinforce the view that secondary symbionts are key modulators of host phenotypes, with outcomes varying by host species and insecticide mode of action.

### 4.2 Suppression of host detoxification machinery

The increased susceptibility in the A-deleted line appears to be driven by a compromised detoxification system. Biochemically, we observed reduced protein levels of MFOs and GSTs following *Arsenophonus* removal. Transcriptomically, this was mirrored by the significant downregulation of KEGG pathways related to “Drug metabolism—cytochrome P450” and “Metabolism of xenobiotics by cytochrome P450.” This pattern parallels findings in *N. lugens*, where antibiotic treatment suppressed P450 and GST activities, leading to increased sensitivity to multiple insecticides (Tang et al., 2021). These findings suggest that *Arsenophonus* modulates host insecticide susceptibility by regulating host detoxification metabolism.

### 4.3 Functional significance of candidate P450 genes

A critical finding of this study is the identification of seven P450 genes (*CYP380C44*, *CYP380C45*, *CYP6J1*, *CYP6CY14*, *CYP6CY21*, *CYP4CJ1*, and *CYP4C1*) that were significantly differentially expressed between the A-infected and A-deleted lines. Among these, *CYP380C44* emerged as a top candidate. Our RNAi validation confirmed that silencing *CYP380C44* significantly increases sulfoxaflor susceptibility, establishing its functional importance. Beyond this study, the biological significance of *CYP380C44* is underscored by its recurrent identification in multi-drug resistance. It has been implicated in resistance to cycloxaprid (Dong et al., 2025) and cyantraniliprole (Zeng et al., 2021), suggesting it is a broad-spectrum detoxification gene. Its downregulation in the absence of *Arsenophonus* implies that the symbiont’s presence is crucial for maintaining high expression levels of this pivotal enzyme.

Notably, the other six P450 genes also have well-documented roles in insecticide resistance, suggesting a synergistic loss of protection: *CYP380C45*, a paralog of *CYP380C44*, has been directly linked to sulfoxaflor and acetamiprid cross-resistance in diverse geographical populations (Wang et al., 2024). Its downregulation likely compounds the effect of reduced *CYP380C44* levels. *CYP6CY14* confers resistance to neonicotinoids (Wu et al., 2018) and the novel insecticide flupyradifurone (Ding et al., 2023). *CYP6CY21* is a known mediator of cross-resistance between diamides and pyrethroids (Peng et al., 2022), while *CYP6J1* is specifically upregulated in sulfoxaflor-resistant strains (Ma et al., 2019a). *CYP4CJ1* is versatile, responding to both host plant gossypol and tannic acid tolerance (Ma et al., 2019b), indicating a role in general xenobiotic adaptation. Similarly, *CYP4C1* homologs are associated with thiamethoxam resistance in other coleopterans (Shi et al., 2021). *Arsenophonus* does not merely affect a single gene but potentially influences a broader regulatory network governing the P450 genes, thereby enhancing tolerance to sulfoxaflor and potentially other chemistries.

### 4.4 Limitations and management implications

While our study highlights the role of *Arsenophonus* and P450s, we acknowledge that antibiotic curing is not perfectly specific. Although *Arsenophonus* was completely eliminated, the treatment caused non-significant but visible declines in other taxa, such as *Bacillus*, *Paenibacillus*, and *Brevibacillus*. Since these genera were abundant in the A-infected line, we cannot rigorously rule out the possibility that antibiotic off-target effects or interactions within the broader microbiome contributed to the observed phenotype. Therefore, while the *CYP380C44* RNAi data proves the gene’s function, the link between *Arsenophonus* and *CYP380C44* regulation remains correlative. Future work using *Arsenophonus* re-infection or specific metabolic inhibition is needed to establish direct causality.

From a pest management perspective, our findings identify symbiont composition as a critical ‘hidden variable’ influencing insecticide susceptibility. The capacity of *Arsenophonus* to maintain elevated P450 levels suggests that high infection rates could facilitate resistance evolution. Therefore, monitoring *Arsenophonus* prevalence in field populations could serve as a practical biological indicator for anticipating sulfoxaflor efficacy fluctuations. Ultimately, these insights highlight the potential for synergistic strategies combining chemical insecticides with symbiont-targeted interventions, offering novel avenues for sustainable Insecticide Resistance Management (IRM) and Integrated Pest Management (IPM).

## Data Availability

The data presented in this study are publicly available. The data can be found here: https://www.ncbi.nlm.nih.gov/bioproject/?term=PRJNA1377489
